# Dientamoeba fragilis: a story of contradictions

**DOI:** 10.1099/jmm.0.002110

**Published:** 2026-01-12

**Authors:** Luke M. Hall, John T. Ellis, Damien J. Stark

**Affiliations:** 1 School of Life Sciences, University of Technology Sydney, Broadway, Ultimo, NSW 2007, Australia; 2 Division of Microbiology, Sydpath, St Vincent’s Hospital, Darlinghurst, NSW 2010, Australia

**Keywords:** diagnosis, *Dientamoeba fragilis*, pathogenicity

## Abstract

*Dientamoeba fragilis* is a gastrointestinal parasite of controversial clinical significance. From its discovery until today, contradictory articles have been published on whether infection is correlated with symptoms, treatment is associated with recovery and whether infection is associated with elevated intestinal inflammatory markers (faecal calprotectin). Additionally, there is no consensus on the infective stage of the lifecycle. Competing theories propose that either *Enterobius vermicularis* ova act as a vector for the transmission of trophozoites or that the cyst stage, which is rarely found, is responsible for infection. In this review, we aim to critique these contradictions to determine if *D. fragilis* should be considered a pathogen in clinical practice. The frequent limitation of studies is challenges in setting up a reliable, healthy control group and the reliability of diagnostic methods. Many studies are opportunistic in design, using samples that have been submitted for routine pathology testing. Even if all pathology tests are negative for infectious agents, the current health status of people who are submitting samples for pathology testing is unlikely to be the best option, just the most available one. Of greater concern is the reliability of some diagnostic methods. Some studies have suggested that at least one of the lab-based real-time PCR assays used for the diagnosis of *D. fragilis* has issues with false positives in human samples. This calls into question much of the evidence that has been published on *D. fragilis* being a commensal instead of a pathogen. As such, *D. fragilis* should be considered a potential pathogen when investigating gastrointestinal illness. Developing better guidelines on determining when *D. fragilis* is the causative agent of symptoms and when to treat are important topics for future research.

## Introduction

*Dientamoeba fragilis* was first described in 1918 as a binucleate amoeba between 8 and 10 µm in diameter [1]. The life cycle of *D. fragilis* includes three stages, all of which can be observed in stool samples at varying frequencies [2]. All three life cycle stages are unified by the presence of a fragmented karyosome in their nuclei. The trophozoite, a binucleated amoeboid cell, is the actively multiplying state found in the gastrointestinal tract and is the most common stage observed in faeces where it takes a rounded form. The pre-cyst stage is a more condensed version of the trophozoite with a typical diameter from 4 to 5 µm. Finally, the cyst stage is differentiated by the presence of a thick outer cyst wall which encases the encysted parasite with a distinctive peritrophic space. The cyst stage is similar in size to the pre-cysts with an overall diameter ∼5 µm [2,4]. These cyst stages are rarely observed in human infections, which has led to debate over how *D. fragilis* is transmitted with faecal–oral transmission in life cycles being depicted to occur via either cysts, trophozoites or trophozoites which have been internalized by pinworm ova (*Enterobius vermicularis*) [5]. Routine diagnosis of *D. fragilis* is achieved by either the microscopy observation of any parasite life cycle stage in permanently stained faecal specimens (typically the trophozoite) or by molecular diagnostic assays which used real-time fluorescence detection [6].

While Jepps and Dobell originally described *D. fragilis* as a harmless commensal [1], this conclusion was questioned a year later when *D. fragilis* was identified in US military officers suffering from bowel complaints [7]. This debate has continued to today, with some researchers proposing that *D. fragilis* is responsible for gastrointestinal complaints, termed dientamoebiasis. The development of a rodent model [4], studies linking infections to gastrointestinal symptoms in children [8] and the identification of virulence factors in the transcriptome [9] have been used to argue the pathogenic potential of this organism. Alternatively, the high prevalence of *D. fragilis* in human populations, up to 71% [10], and longitudinal studies showing no statistical association between children testing positive for *D. fragilis* and gastrointestinal symptoms [11] cause some researchers and medical professionals to argue that *D. fragilis* should not be considered when investigating the cause of gastrointestinal complaints. While it is debated if *D. fragilis* is a pathogen, it is distributed globally.

Since its characterization in 1918, the taxonomy of *D. fragilis* has changed dramatically. Originally identified as a member of the Entamoebidae due to apparent morphological similarity with the three *Entamoeba* species known to occur in the human bowel: *Entamoeba coli*, *Entamoeba nana* (now known as *Endolimax nana*) and *Entamoeba histolytica* [1]. Subsequent studies with microscopy revealed morphological similarity with *Histomonas meleagridis* [12], which later led to the creation of a new family, Dientamoebidae [13,14]. Molecular investigations of the SSU rDNA confirmed that *D. fragilis* and *H. meleagridis* were related, and they shared a common evolutionary history with trichomonads [15]. The phylogeny of Parabasalia species has subsequently been supported by additional molecular characterization of *Rpb1*, actin and elongation factor-1*α* [16,17]. A revised classification scheme for *D. fragilis* (Table 1) is based on morphological analysis and molecular characterization of the ITS and SSU rDNA [18]. Boscaro *et al*. [19] further updated the taxonomic classification of *D. fragilis* by splitting the previous Tritrichomonadidae class [18] into four: Tritirchomonadea (redefined), Dientamoebea, Simplicimonadea and Monocercomonadea. The basis of this change is that Tritrichomonadidae was a paraphyletic assemblage with no reliable morphological trait that united them [19].

**Table 1.** Two modern taxonomic classifications of *D. fragilis* Based on morphological analysis and molecular characterization of the ITS and SSU rDNA [18,19].

Taxonomic rank	Classification [18]	Classification [19]
Phylum	Parabasalia	Parabasalia
Clade	–	Cadamassta
Class	Tritrichomonadidae	Dientamoebea
Order	Trichomonadida	Dientamoebida
Family	Dientamoebidae	Dientamoebidae
Genus	*Dientamoeba*	*Dientamoeba*
Species	*D. fragilis*	*D. fragilis*

The current research data on *D. fragilis* contains many contradicting studies. This review aims to critically review this research to determine if *D. fragilis* should be considered a pathogen and provide an overview of the current state of research on this gastrointestinal parasite.

## Diagnosis

The diagnosis of *D. fragilis* is completed using either microscopy with a permanent stain or PCR on faecal specimens. Correct and timely sample preparation for both approaches is essential for success as *D. fragilis* is known to lose viability and degrade quickly when passed from the host. While antigen detection assays have been developed for *Cryptosporidium*, *Giardia* and *E. histolytica* [20], no commercial immunoassay has been developed for *D. fragilis*; instead, the development of molecular PCR methods has been favoured. Immunoassays are a poorly researched option for *D. fragilis* limited to one study in 1993 which demonstrated the potential for developing immunoassays for *D. fragilis*, producing an indirect fluorescence assay (IFA) using an antibody produced in a rabbit [21]. No false positive results were observed. Seven out of nine *D. fragilis* samples were successfully detected, while the two remaining samples had an indeterminate IFA result, explained by low trophozoite presence on stained smears. No further publications on diagnostic immunofluorescence assays have been published. Additionally, there is one study on the use of matrix-assisted laser desorption/ionization-time of flight MS as a tool for detecting *D. fragilis* in simulated stool samples [22]. Fifteen peaks were found to be able to discriminate between *D. fragilis* and the culture medium (Robinson’s Media); only 6 were found in all 14 clinical isolates tested [22]. Differences in protein expression may be the result of different expressions based on culturing conditions. It was not investigated if these peaks could differentiate between *D. fragilis* and other protozoa, causing the specificity of this method to be unknown. To our knowledge, this technique for detecting *D. fragilis* has yet to be routinely applied in any pathology setting. Routine screening for *D. fragilis* is not universally completed in the standard microbiology diagnostic workflow since the screening employed reflects the priorities of a laboratory/medical system. Since *D. fragilis* is not yet universally accepted as a pathogen, many pathology laboratories do not routinely test for it.

### Microscopy

Microscopy with a permanent stain is still considered the gold standard for *D. fragilis* diagnosis, even though many pathology laboratories in resource-rich settings presently use molecular methods. Direct examination of faecal specimens is not recommended as the trophozoites will appear as refractile round forms without any nuclear detail, even when using an iodine preparation. This detail is insufficient for identification. There are multiple permanent stains that can be used for the diagnosis of *D. fragilis*: trichrome, iron-haematoxylin, chlorazol black stain and Giemsa and modified Ziehl-Nielsen [23].

**Fig. 1.**
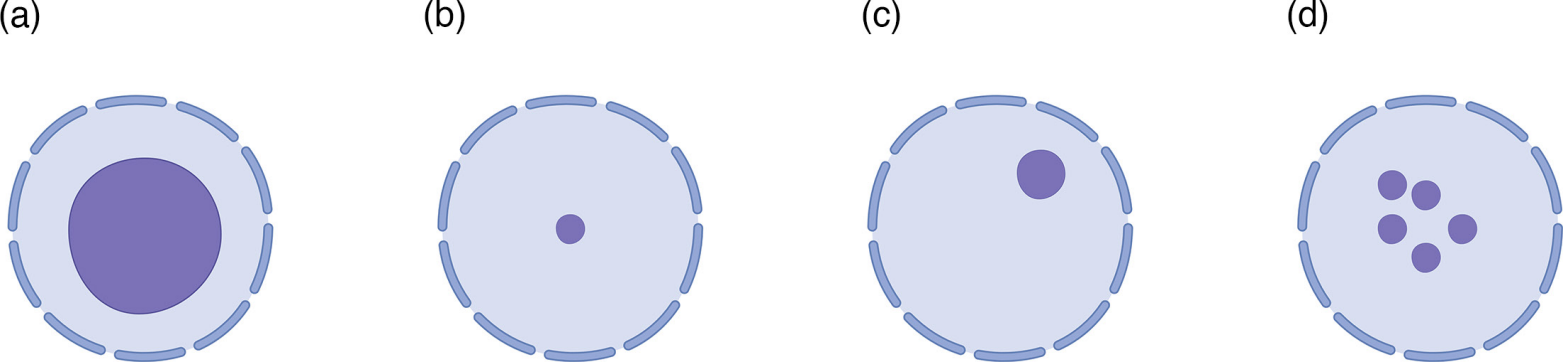
Nuclear karyosome structure of parasites found in stool samples. Structures (a)–(c) depict the common karyosome morphology of amoeba in humans: (a) depicts a fisheye karyosome, (b) a compact, centrally located karyosome and (c) an eccentric karyosome. Depicted in (d) is the fragmented karyosome of *D. fragilis*, a key feature for its definitive morphological diagnosis. Created in https://BioRender.com*.*

For effective microscopic diagnosis of *D. fragilis,* quickly fixing the faecal sample in a fixative that is compatible with the staining procedure is essential. Collected faecal samples are frequently used for other diagnostic approaches, culturing or PCR, and fixed samples are not compatible with these approaches. This means that fixation of a portion of the sample needs to be completed in the laboratory when it arrives to prevent multiple samples being collected. Quickly fixing samples is more important for *D. fragilis* than any other intestinal protozoa, as human samples frequently only have the fragile trophozoite stage in them, which degenerates quickly [2]. This degradation decreases diagnostic sensitivity and specificity. Additionally, it is recommended that at least three different samples over 10 days are collected for microscopy examination for the best results [6]. For the diagnosis of *D. fragilis* using microscopy, the expected size of the cell is ~10 µm, and the key morphological feature is the fragmented karyosome of the nucleus. In every described life cycle stage (cyst, pre-cyst and trophozoite), *D. fragilis* has one or two nuclei with the fragmented karyosome [2]. While the overall morphology of *D. fragilis* may appear similar to some amoeba in human stool samples, they can be easily differentiated through identifying the type of karyosome (Fig. 1). *D. fragilis* will have a fragmented karyosome (Fig. 1d), while the amoeba will have either a fish eye, compact centrally located or eccentric karyosome (Fig. 1a–c).

### Culture

Clark and Diamond [24] and Stark *et al*. [25], in their previous reviews, have outlined the various culture methods that are available for *D. fragilis*. As with microscopy methods, the cultivation of *D. fragilis* requires fresh faeces to ensure the greatest likelihood of success [26]. *D. fragilis* grows best in a biphasic culture system of Loeffler’s medium in combination with a modified Earle’s balanced salt solution supplemented with cholesterol and ferric ammonium citrate under microaerophilic conditions at 41–42 °C [27,28]. In our experience, *D. fragilis* can also be grown using PBS and rice starch as a simple overlay with a Loeffler’s slope. Cultivation of gastrointestinal parasites has a limited role in diagnostic pathology laboratories and should not be relied upon for diagnostic purposes, but it does have important applications in many research projects [24]. The main limitation regarding the use of culture for the diagnosis of *D. fragilis* is the need for fresh samples which often cannot be guaranteed. Therefore, negative culture does not confirm the absence of a *D. fragilis* infection. The duration of cultures can vary greatly; some can be sustained for years, while others will last only a few days. Cultures are also slow to establish and the peak numbers are very variable.

### PCR

Both conventional PCR and real-time PCR (qPCR) assays have been developed for the detection of *D. fragilis* that have previously been summarized [25]. Both conventional PCR and qPCR assays have been developed. Commercial assays use qPCR technology due to the enhanced speed in which the result can be generated, as gel electrophoresis is not required since fluorescence is detected in real time. Most commercial assays are multiplexed to allow for the screening of multiple targets (Table 2). Conventional assays still have many uses in research settings as the generated products are larger and can provide genotype information when sequenced, depending on the locus used [29]. Assays using PCR methods are more sensitive for the detection of *D. fragilis* than microscopy [23,30].

**Table 2.** Commercially available and most common lab-based real-time PCR (qPCR) assays used for the diagnosis of *D. fragilis*

Assay	Assay type	Company
AllPlex GI-Parasite Assay	Multiplex qPCR	Seegene
EasyScreen enteric parasite detection kit	Multiples qPCR	Genetic Signatures
RIDA ®GENE Parasitic Stool Panel I	Multiplex qPCR	R-Biopharm AG
LightMix Modular Assay	Multiplex qPCR	Roche
Gastrointestinal Parasites	Multiplex qPCR	AusDiagnostics
VIASURE: Gastrointestinal Panel II	Multiplex and Monoplex qPCR options	Certest Biotec
Dientamoeba fragilis PCR Kit	Monoplex qPCR	ALPCO
Gastrointestinal Parasite Panel by PCR	Multiplex qPCR	ARUP Laboratories
Lab-based	Monoplex qPCR	[37]
Lab-based	Monoplex qPCR	[116]

Multiple commercial assays have been used for the detection of *D. fragilis* in published research [30,33]. Comparisons of two of these techniques on clinical samples revealed discrepancies in the results and identified differences in the performance of one assay, which were attributed to the laboratory equipment used and non-specific/non-target amplification [34,35]. This highlights the importance of optimizing and validating the results generated by these assays when integrating them into a diagnostic workflow. This should include consideration of whether positive results need further assessment to confirm their accuracy, which could be achieved using melt curve analysis for compatible assays [36]. Additionally, these discrepancies call into question the reliability of the data generated in studies using the lab-based assay developed and primarily used in Europe in studies describing *D. fragilis* as a commensal [11,37]. Since samples are falsely determined as positive for *D. fragilis*, this impacts studies that investigate the correlation with symptoms, inflammation markers and recovery post-treatment.

In addition to their use in human diagnostics, commercial and lab-based qPCR assays have been increasingly used to screen a range of animal species to gain a better understanding of the zoonotic potential of *D. fragilis* [35,36, 38, 39]. While these molecular methods do allow for increased ease of screening, it is important to consider the potential for cross-reactivity with other related Parabasalid species that are found in species other than humans. For the assays that have been assessed (EasyScreen and two lab-based assays), cross-reactivity has been detected against *Tritrichomonas foetus*, *Pentatrichomonas hominis* and *Simplicimonas* sp. [35,36, 40]. This means that while qPCR can be used for high-throughput screening, additional sources of evidence including microscopy or sequencing are required to confirm *D. fragilis* infection in a new host species.

## Epidemiology

### Transmission

**Fig. 2.**
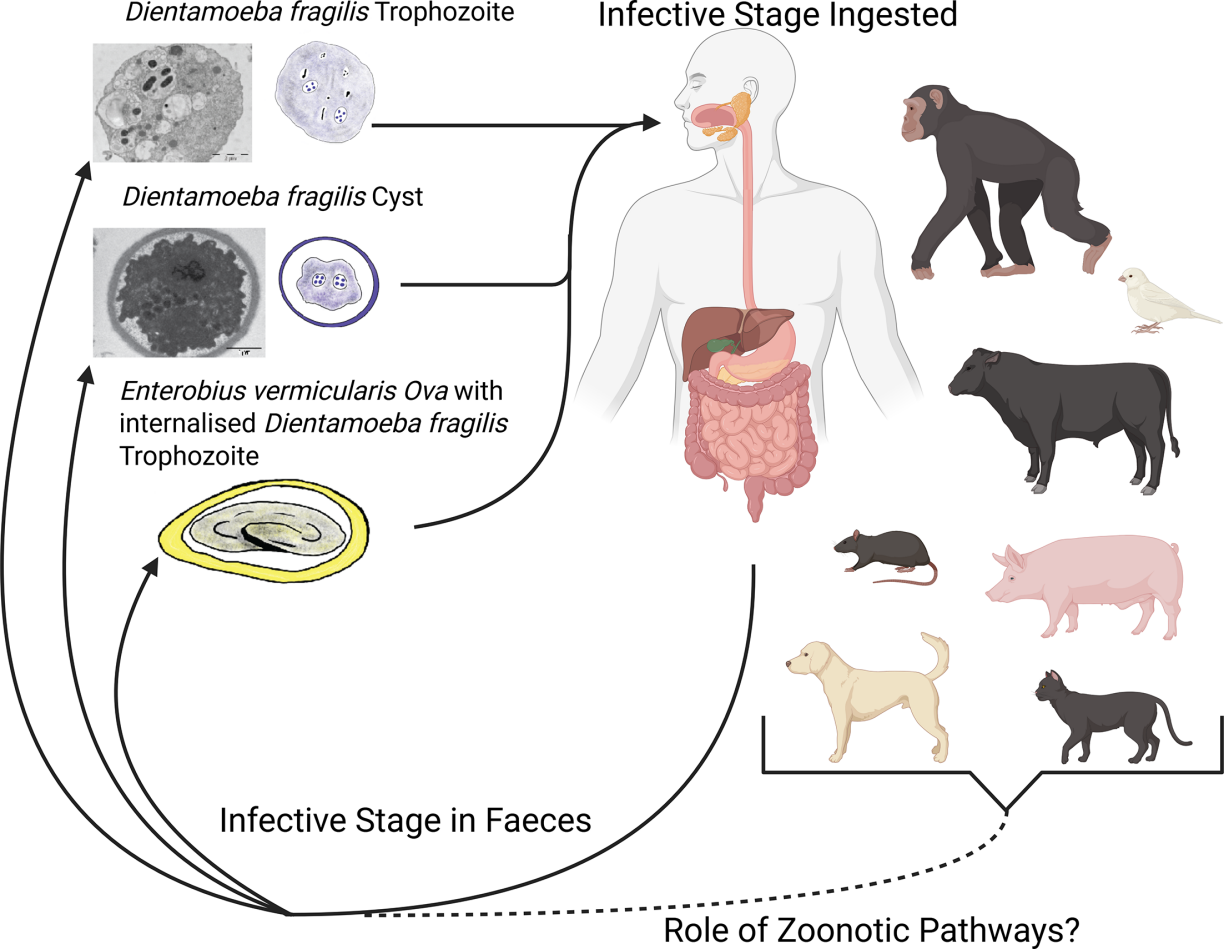
Life cycle and hypothesized transmission pathways of *D. fragilis*. *D. fragilis* replicates by binary fission in the lumen of the large intestine and is shed in the stools of humans. The transmission of *D. fragilis* between hosts is not well understood. It is possible that direct faecal–oral transmission by either trophozoite or cyst stages, or transmission via *E. vermicularis* eggs occurs. The detection of *D. fragilis* in the faeces of cats, dogs, pigs, cows, rats, budgerigars and non-human primates indicates a potential role for zoonosis in human infection. Created in https://BioRender.com*.*

There are three theories about how *D. fragilis* transmits between hosts: first, direct faecal–oral transmission by trophozoite; second, faecal–oral transmission via the cyst stages; and finally, transmission via *E. vermicularis* eggs (Fig. 2). Since its discovery, the trophozoite stage is known to be fragile [1], hence the species name ‘*fragilis*’. Amoeboid forms observed in active cultures and inside the host quickly become rounded then degreed when exposed to external environments [1]. Therefore, the trophozoite is unlikely to survive for long under the environmental conditions required to transmit between hosts [3]. In the human population, the trophozoite is the predominant stage seen by microscopy in faecal specimens [2]. The incongruity of this fragile stage being responsible for the transmission of *D. fragilis* led to the development of the cyst stage and helminth vector models of transmission. Recent evidence has demonstrated that these trophozoites are unable to survive acidic conditions of a new host’s stomach [3], further highlighting the importance of these alternative theories of transmission.

Initially, there was no identified cyst stage of *D. fragilis*. The absence of this life cycle stage led researchers to propose that *D. fragilis* transmission occurs using a helminth vector, *E. vermicularis* [41]. In support of this theory, multiple studies have identified correlations between *D. fragilis* and *E. vermicularis* infection [42,43]. Additionally, *D. fragilis* DNA was detected inside surface-sterilized *E. vermicularis* ova isolated from co-infected hosts [44,45]. A limitation of this model is that the presence of DNA does not confirm the presence of viable *D. fragilis* cells. No studies have been completed to determine if ingestion of *E. vermicularis* ova with internalized *D. fragilis* can infect a new host with *D. fragilis*. Additionally, *E. vermicularis* is a human-specific helminth rarely detected in other primates [46]. Since *D. fragilis* has been found in many non-primate hosts (cats, dogs, cows and pigs), if *E. vermicularis* were required for the infection of a new host, these species would all be dead-end hosts of *D. fragilis* since the parasite would be unable to use the ova of *E. vermicularis* to transmit to another host. This raises the question of whether it is likely for *D. fragilis* to have so many dead-end hosts in the case of helminth-mediated transmission. Notably, evidence proving one theory of transmission does not disprove the other theories. Helminth-mediated transmission could be exclusive to human–human transmission. Zoonotic transmission and transmission between animals may occur via the cyst stage.

Even today, after the discovery of the cyst stage in a rodent model [4], there have been only a few reported instances of cyst or pre-cyst stages of *D. fragilis* in human clinical samples (5 true cysts from 500 patient slides) [2,47]. While the cyst stage is the most common stage known to be responsible for the transmission of gastrointestinal parasites, the low prevalence of cyst stages in humans when compared to the rodent model is a major hurdle for this theory. Zoonotic transmission of *D. fragilis* using the cyst stage is possible, but all studies on animals have either not used microscopy or have not reported on the cyst stage for the few that did. Of particular interest is the recent detection of *D. fragilis* in rats trapped in parks and the sewer system in Barcelona (Spain), raising the possibility that rodents are a significant vector for this parasite [48]. Unfortunately, this study detected the presence of *D. fragilis* only using PCR and did not provide any descriptions of the parasitic stages found in the specimen. Previous observations from laboratory models detected the cyst stage of *D. fragilis* in rodents [4]; this detection of natural infections of rats with *D. fragilis* [48] supports the potential of rodents (rats) as vectors for *D. fragilis*, potentially via the cyst stage.

In addition to rats, *D. fragilis* has also been detected in cats, dogs, cows, pigs, goats, rabbits, sheep, guinea pigs, budgerigars and non-human primates [36,38, 49,51]. The data supporting cows, pigs, budgerigars and non-human primates as hosts for *D. fragilis* is strong, as they all included sequence data to support detection using microscopy and/or qPCR [49,51]. For cats, dogs, goats, rabbits, sheep and guinea pigs, only qPCR data was used [36,38]. Since qPCR assays have been shown to cross-react with non-target species, additional evidence is needed to confirm these animals as hosts for *D. fragilis* [35,36]. One way this was done was by using a melt curve analysis of the qPCR product to demonstrate that the positive results in cats and dogs were consistent with *D. fragilis* [36]. While our understanding of the role that zoonosis plays in human infection is still developing, one study found the same genotype of *D. fragilis* in humans and pigs from the same farm [51]. This highlights the need to consider zoonosis when investigating infection pathways for *D. fragilis*.

### Prevalence

The reported prevalence of *D. fragilis* varies greatly depending on the diagnostic techniques used, geographical location and the subset of the population being investigated. Reported prevalences for populations including all age groups vary from 0.04% [52] to 43% [10]. Unlike most other gastrointestinal parasites, *D. fragilis* is reported to have the highest prevalence in countries with developed economies and healthcare (Table 3). However, this could be due to the use of diagnostic methods with varying sensitivity due to the lack of a global standard. There are two main diagnostic techniques used for the detection of *D. fragilis*: light microscopy and PCR (favoured in recent years). Multiple studies have shown that detection sensitivity for *D. fragilis* varies greatly depending on the technique used [23]. One study of 575 samples reported detection in 5.0% and 14.8% of individuals by microscopy and PCR, respectively [23].

**Table 3.** Reported prevalence of *D. fragilis* in human populations using real-time PCR assays (qPCR). A large variation in the prevalence has been shown even when only comparing qPCR assays, though the commercial makeup of these assays does vary*.*

Location	Population	Size *N*	Assay type	*D. fragilis* prevalence (%)	Source
Denmark	Clinical samples	22,484	qPCR	43	[10]
Israel	Children	36,008	qPCR	32.5	[117]
Israel	Clinical samples	138,415	qPCR	29	[118]
Israel	Clinical samples	27,918	qPCR	22.3	[70]
Turkey	Clinical samples	200	PCR	16	[56]
Luxembourg and France	Clinical samples	2034	qPCR	13.1	[119]
Australia	Clinical samples	254	qPCR	11	[35]
Italy	Clinical samples	864	qPCR	9.1	[29]
Indonesia	School children	338	qPCR	4.1	[120]
Ghana	HIV patient, health controls and children	1,569	qPCR	0.8	[121]
USA	Clinical Samples	4,804	qPCR	0.6	[122]

### Risk factors

The main risk factor for *D. fragilis* is age. Young children (5–9 years) are the age group at highest risk of being infected with *D. fragilis,* which is explained by the poor hygiene practice of young children [10]. The age distribution of infection by *D. fragilis* follows the same bimodal trend as many other gastrointestinal parasites. The second peak is in the primary contacts of these infected children (40–44 years, predominantly females).

Additional risk factors include proximity to animals and international travel. *D. fragilis* has been detected in many host species other than humans. This would make it logical to conclude that contact with animals would increase the risk of infection. While studies have found *D. fragilis* in cats and dogs [36,38], studies that attempted to assess if contact with domestic pets did increase the risk of infection found varied results, with two concluding it had no effect [11,38] and one that it did [53]. Increased risk in association with farm animals/rural areas has been noted in two studies [38,53]. This indicates that pets may not be the animals’ most likely source of infection, potentially due to hygiene practices maintained by pet owners and the exposure of farm animals to a wider range of possible sources of infection including wild animals.

International travel is a risk that is frequently identified to cause elevated rates of *D. fragilis* infection. Denmark has the highest reported prevalence of *D. fragilis* in humans, yet studies in the country identify international travel as a risk factor for testing positive for *D. fragilis* [11]. A study in the Czech Republic also found an increased risk of *D. fragilis* in people who travelled internationally, particularly those who travelled outside Europe [38]. Typically, an increased risk of infection due to international travel is associated with travel to areas where a disease is more prevalent. As such, this strengthens the argument that the higher infection rates reported in countries with developed economies and healthcare settings may be due to better diagnostic methods that are more routinely used instead of a genuine higher rate of infection in these countries.

### Molecular diversity

One of the theories for the differential clinical presentation of *D. fragilis* is that there is undescribed diversity within the species across a spectrum of pathogenicity. Currently, there are only two described genotypes of *D. fragilis* differentiated by sequencing a portion of the 18S rDNA gene based on 10 nucleotide differences in a 558 bp PCR product (2% difference) [54,55]. Genotype 1 is most commonly detected in humans [25]. Recent studies on the genotypes of *D. fragilis* use the 18S rDNA to identify genotype 1 with minimal variability [29,56]. This data continues to support the concept of *D. fragilis* as a clonal species, though few genetic markers have been analysed. As such, the strength of evidence supporting this is to be limited, and analysis using additional markers or genome-level comparisons is likely to change this understanding.

A novel multi-locus sequence typing (MLST) protocol was developed using metagenomic data of the gut microbiota of people infected with *D. fragilis* [57]. Six loci were used targeting RNA polymerase, kinase, peptidase and laminin A genes. *D. fragilis* samples from Europe, Australia and Brazil were analysed using this technique, which resulted in the identification of one main multi-locus group (MLG) dispersed across all geographical locations and six MLGs radiating out from it based on point mutations [57]. When this assay was used to genotype samples from Turkey, different haplotypes were only found using one locus [56]. The limitation of this MLST assay is that a limited number of markers were used and were identified due to detection in a metagenomic approach instead of screening for genes with increased variance between isolates [57]. As such, the genes that were selected were those that presumably have multiple copies in the genome, but not necessarily those that are rapidly mutating. Nothing is known about the rate of evolution of these genes in *D. fragilis* and their suitability for genotyping populations. Not all genes are suitable for detecting molecular diversity within a species in all cases. To truly be able to determine if the spread of *D. fragilis* is clonal, a better understanding of which genes are evolving is needed. This could be effectively done when a robust method for sequencing the genome of *D. fragilis* is developed, as this would remove/decrease the bias towards more abundant genes.

## Pathogenicity

Since its discovery, the pathogenicity of *D. fragilis* has been debated. The development of rodent models, where infected animals developed symptoms of gastrointestinal illness, has gone a long way to fulfilling Koch’s Postulates [4,58]. However, current research is still highly conflicted because the pathophysiology of dientamoebiasis is not well understood. Elevated faecal calprotectin, a marker for inflammation, is one conflicted area of research. The study that links elevated faecal calprotectin to *D. fragilis* infection compared samples of symptomatic *D. fragilis* cases to healthy uninfected controls [59]. In contrast, another study found no correlation in calprotectin levels based on *D. fragilis* infection status regardless of whether the samples belonged to the ‘health control’ and ‘infected’ cohorts [60]. Studies have investigated the correlation of clinical symptoms with infection of *D. fragilis*, effectiveness of treatment at the eradication of symptoms and molecular pathogenic pathways attempting to determine the pathogenicity of *D. fragilis*. Other studies have investigated parasite load as a marker for pathogenicity with contradicting results [61,62].

### Correlation with clinical symptoms

When discussing whether *D. fragilis* is a pathogen, many studies investigate whether infection is correlated to disease. Some studies have found links between *D. fragilis* infection and gastrointestinal symptoms: abdominal pain, anal itching, bloating and diarrhoea [8,23, 63, 64]. Additionally, some of these studies have reported effective treatment of *D. fragilis* using paromomycin [23] and secnidazole [63], indicating that *D. fragilis* was the causative agent. However, other studies refute these conclusions, finding no links between *D. fragilis* infections and GI symptoms in day-care centres [11,60]. Additionally, these studies report that the prevalence of *D. fragilis* is higher in participants without symptoms than in those with symptoms [60,65]. While these studies provide insight into whether the parasite can be linked to disease, it fails to confirm a causative link, which is essential if *D. fragilis* is to be widely recognized as a pathogen.

The high proportion of *D. fragilis* infections in asymptomatic individuals is not a reason to dismiss this protozoan as a pathogen. For an organism to be considered a pathogen, it is not required that all infections cause a symptomatic infection. *Giardia duodenalis* is a gastrointestinal protozoan parasite that is the causative agent of giardiasis characterized by the following symptoms: diarrhoea, epigastric pain, greasy stools, flatulence, nausea and weight loss [6,66]. One study investigating protozoa in 209 non-native children in Italy determined that 69.8% (30 cases) of *Giardia* and 78.5%(73 cases) of *D. fragilis* infections were asymptomatic [67]. Similarly, other studies have also reported asymptomatic infections of *Giardia* at higher rates than symptomatic infections [68,69].

Recently, multiple studies using molecular diagnostic methods have concluded that *D. fragilis* detection is not associated with clinical disease [62,70]. These large retrospective studies that have recently been published are limited and undermined in their integrity by their defined control group. A person who is submitting a faecal sample to a pathology lab for parasite investigations, whether any parasites are present, is unlikely to be a good healthy control in any study. Control patients in one study reported at least one gastrointestinal symptom in 66.7% of participants [62]. These samples/individuals are used in these retrospective studies as the samples have already been collected. Just because they were parasite negative or negative for all infectious agents tested for use in the laboratory does not rule out non-infectious causes of diarrhoea. These studies need to review the clinical notes and other investigations completed to remove individuals with non-infectious diarrhoea, such as food intolerances, irritable bowel disease and medication-induced diarrhoea. Failure to do so will cause the healthy control to include people with gastrointestinal issues not caused by parasitic infections and bias studies towards not seeing a difference in the rate of gastrointestinal symptoms in the so-called ‘healthy’ population when compared to those infected with *D. fragilis*.

### Molecular pathogenic pathways

While it is crucial to identify pathogenic pathways to confirm *D. fragilis* as a causative agent of gastrointestinal illness, there is presently a limited number of studies in this area. To date, there is only one published transcriptome of *D. fragilis,* which aimed to identify the virulence factors of *D. fragilis*. In this transcriptome, virulence factors similar to those of known pathogenic parasites (*Trichomonas vaginalis*, *T. foetus* and *E. histolytica*), including cysteine peptidases and saposin/amoebapore family proteins, were identified [9]. Of particular significance, five transcripts were similar to a known cytotoxic cysteine proteinase from *T. vaginalis*. In *Trichomonas* sp. and *T. foetus*, these cysteine peptidases are capable of degrading host proteins including collagen (I, III, IV and V), gelatin, immunoglobulin (IgG and IgA) and complement component 3 [71,75]. [9] hypothesized that *D. fragilis’s* cysteine peptidase serves a similar function to their counterparts in *Trichomonas* sp. and *T. foetus*, being a possible mechanism for pathogenic characteristics [9].

### Irritable bowel syndrome

*D. fragilis* has been a suspect in the aetiology of irritable bowel syndrome (IBS) [25,76]. Some studies report a weak association between *D. fragilis* and IBS [76,78]. However, a recent systematic review of 17 studies (5,882 participants) on the association between *D. fragilis*, *Blastocystis* sp. and IBS found that while there was a significant association with *Blastocystis* sp., the analysis on *D. fragilis* was not [79]. A subsequent study from Turkey also found no association [80]. A study in Denmark on the treatment of 25 IBS patients also with *D. fragilis* using metronidazole (23) and tetracycline (2) determined that there was no significant improvement of IBS symptoms [81]. Fifteen patients were reported as having *D. fragilis* eradicated, but only seven had any clinical improvement [81]. This indicates that *D. fragilis* may have a limited role, if any, in the aetiology of IBS. However, this was only a small study and infections treated using metronidazole can be prone to relapse, so further epidemiological and case-controlled studies are required [82,83].

### Pathohistological changes

There is a limited number of studies that have reported on histopathological changes due to *D. fragilis* infection. Early studies reported the presence of *D. fragilis* in the appendix in 0.7–4.5% of cases studied [84,86]. Trophozoites were not observed to invade the tissue; however, they did ingest blood cells, and marked fibrosis of the appendix was noted [84,86]. Other early studies detected *D. fragilis* in a case of ulcerative [87] and eosinophilic colitis [88]. On the association between *D. fragilis* and eosinophilia, early observations about *D. fragilis* causing eosinophilic colitis are further supported [89,90]. In more recent studies on mice, increased inflammation of the intestine was reported [4,58]. Overall, the lack of data limits our ability to confirm the histological impacts of *D. fragilis*, though other methods (faecal calprotectin) have been used to investigate the inflammatory response of the gastrointestinal system in response to infection.

### Faecal calprotectin

Two studies have investigated the relationship between faecal calprotectin, a mark of intestinal inflammation and the carriage of *D. fragilis* in humans and one in mice. The logic behind these studies is that if *D. fragilis* is a pathogen, then the immune response against it will cause inflammation, resulting in an increased calprotectin concentration, and if it is a commensal, then this would not be observed. The study on laboratory-infected mice showed a twofold increase in faecal calprotectin for the *D. fragilis*-infected mice (*n*=16) when compared to the control (*n*=2) [4]. However, in humans, the studies arrived at different conclusions due to arguably biassed study designs; one reported a correlation between increased calprotectin and *D. fragilis* infection [59], and the second found no correlation [60]. The study that found no difference utilized samples from asymptomatic controls and individuals with persistent or recent gastrointestinal symptoms. In the control group, *D. fragilis* was detected more frequently (71%) than the symptomatic group (45%). Statistical analysis of the faecal calprotectin concentration between samples with or without *D. fragilis* found no significant difference [60]. The conclusion from the analysis that *D. fragilis* does not cause elevated faecal calprotectin is invalid since ~75% of the samples in the group with no *D. fragilis* originated from the symptomatic cases set of samples, which while not being infected with *D. fragilis* are likely to have an underlying cause of their symptoms that may elevate their faecal calprotectin levels. Aykur et al. [59] used an improved study design that established three groups of samples; symptomatic patients with *D. fragilis* detected, symptomatic patients without *D. fragilis* detected and healthy controls without infections or symptoms. In this study, *D. fragilis* was associated with significantly higher faecal calprotectin levels than the other two groups. The main issue with this study is that it ignores asymptomatic infections of *D. fragilis*. Ideally, any future study would also include an additional asymptomatic group with *D. fragilis* detected to increase confidence in the observation of elevated faecal calprotectin with symptomatic *D. fragilis* infection. Alternatively, faecal calprotectin could have potential as a biomarker to indicate when cases of *D. fragilis* should be treated if there is a significant difference between the asymptomatic and symptomatic samples with *D. fragilis*.

## Management

### Prevention

All three proposed methods of transmission agree that *D. fragilis* is transmitted between hosts by the faecal–oral route; the only argument is over the infective stage. This means that the traditional measures of good hand hygiene and faecal waste management used to prevent the spread of gastrointestinal pathogens are applicable to this species. Washing hands thoroughly with soap and using a clean towel to dry them before food preparation and eating, after using the toilet and changing diapers is recommended. Additionally, adequate disposal measures for faecal material are needed to prevent contamination of food and water to help prevent the spread of *D. fragilis* [6]. The detection of *D. fragilis* in ready-to-eat packaged salads highlights the need to wash fresh fruit and vegetables [91].

### Treatment

While it is still debated over whether *D. fragilis* causes gastrointestinal symptoms, studies do not normally associate the infection with severe complications. The impact on patients with compromised immune systems is not well understood. Studies have shown no increased rate of infection of *D. fragilis* in immunodeficient groups [92,93]. Patients with *D. fragilis* have been reported to show improvement of gastrointestinal symptoms after treatment [23,94]. Therefore, it is recommended to treat symptomatic *D. fragilis* infections when other diagnoses have been eliminated. Treatment options include metronidazole, paromomycin, iodoquinol, tetracycline, doxycycline and secnidazole [66,82]. When determining whether to provide antiparasitic treatment for an infection of *D. fragilis*, it is important to assess the severity and duration of symptoms and other possible clinical causes and balance the potential benefit with the side effects of the medication being selected for use.

While no large case-controlled or randomized clinical studies have been completed to assess the effectiveness of these treatments, evidence on their efficacy has been investigated through retrospective/longitudinal studies and one small randomized clinical trial. In the randomized clinical trial, 96 children were treated with either metronidazole or a placebo [83]. They did not observe any clinical improvement in the metronidazole-treated patients compared to those given the placebo. It is important to point out that the success rate of metronidazole treatment at 14 days was 62.5%, which declined to 24.9% at the 56-day follow-up, indicating that the treatment used was ineffective. The drug choice in this study was limited to metronidazole, being the only drug registered for the treatment of *D. fragilis* in Denmark. Other comparative studies between paromomycin and metronidazole indicate that paromomycin is a more effective treatment for clearing *D. fragilis* and resolving symptoms, being effective in 80–100% of cases [23,94, 95]. Therefore, it is recommended to use paromomycin as the treatment of choice for the eradication of *D. fragilis*. Previous reviews on the treatment options for *D. fragilis* showed that studies have reported a wide range of success when using previously mentioned treatment options with efficacy ranging from 12.5% to 100% [82,96]. This means that when treating patients, symptoms not resolving could be due to treatment failure and therefore this possibility should be considered when following up with the patient.

## Microbial interactions

In recent years, there has been increasing recognition of the role that the gut microbiome and microbial interactions play in the overall health of individuals [97,100]. The human microbiome is a dynamic community of microbes that colonize the gastrointestinal system. Research on the microbiome has rapidly advanced due to the development of better sequencing technology and community interest. *Bacteroidetes* and *Firmicutes* are two groups of bacteria of particular interest. In patients with obesity, the balance of these two groups favours *Firmicutes* compared to lean controls, and after diet therapy, this balance shifts back towards *Bacteroidetes* [101]. Another area of interest is the gut–brain axis, which considers the potential for the gut microbiome and people’s moods to be linked and highlights the potential of altering the microbiome to improve cognition in patients with major depressive disorder [102]. The potential of faecal microbiota transplantation to have a curative effect on IBS (subtype D) was demonstrated in a case-controlled study of nine patients for whom IBS severity score reduced from 290 to 144 after three treatments [103]. This improvement was linked to increased species richness within a single sample (alpha diversity), with *Bacteroidetes* and *Firmicutes* being the most abundant [103]. High bacterial richness is often associated with healthy gut microbiomes, while a decline in species diversity has been associated with disease, including irritable bowel disease and type 2 diabetes [104]. Studies like these on the microbiome have been opening many new avenues of research.

Microbial parasites (protozoan parasites) have evolved to live in complex microbial environments, exposed to a range of bacteria, archaea, viruses and microbial eukaryotes (protozoa and fungi) in addition to their host [100]. The various interactions in this environment range from commensal to parasitic [105,106]. The interactions between microbial parasites and the rest of the microbial environment have the potential to influence the host-parasite disease dynamics. Many of the mechanisms through which this modification occurs and the effect that this has on human health are poorly understood for most parasitic species [105]. An example of one known mechanism is how *T. vaginalis* virus (TVV), *Mycoplasma hominis* and *T. vaginalis* interact to alter pathogenesis. While *T. vaginalis* causes tissue damage on its own, TVV and *M. hominis* can act with it in synergy to increase inflammation [107,108]. In these cases, treatment of *T. vaginalis* with metronidazole has been shown to release large amounts of TVV, further activating the immune system [109]. While no virus of the *Totiviridae* family has been sequenced from samples of *D. fragilis*, virus-like particles have been found in trophozoites grown in culture using transmission electron microscopy [110]. Due to the interaction between TVV and *T. vaginalis*, this observation identifies one mechanism through which the microbial environment could alter the pathogenicity of *D. fragilis*. Highlighting an area of research that could potentially help to explain why some studies identify *D. fragilis* as a pathogen and others as a commensal.

Investigation into changes of the gut microbiome in association with *D. fragilis* infection is a new area of research with only a few studies completed. The gut microbiome is another area of research that may help explain the variation in observations on the clinical significance of *D. fragilis* by identifying patterns in microbiomes that explain differences in clinical presentations. One study found no change in the microbiome between 19 children infected by *D. fragilis* with symptoms and 19 healthy controls [111]. Of the 19 healthy controls, 16 were PCR positive for *D. fragilis*; therefore, the researchers concluded that the bacterial microbiota may not play a role in the presence of clinical symptoms [111]. Two additional studies have reported higher alpha diversity associated with *D. fragilis* infection due to an increase in the abundance of bacterial species belonging to *Ruminococcaceae*, *Rikenellaceae*, *Clostridiales vadin* BB60 group and *Christensenellaceae* families [112,113]. Additionally, there is decreased heterogeneity in patients infected with *D. fragilis* compared to controls [113]. Due to the limited number of studies, caution is needed to interpret these results. While increased alpha diversity is frequently associated with healthy microbiomes [104], studies of a related protozoa, *T. vaginalis*, have revealed associations with both increased and decreased bacterial diversity, both related to disease [100]. When this diversity is excessively increased in association with *T. vaginalis,* such cases progress to cause bacterial vaginosis, a pro-inflammatory disease of the urogenital tract [100,114]. This change in bacterial diversity is associated with the loss of lactobacilli and an increase in *Mycoplasma*, *Parvimonas*, *Sneathia* and other anaerobes [114]. The previously mentioned association between IBS and *D. fragilis* infections [25,76] is one area of research that could benefit from considering the impacts of changes in the microbial environment. Changes to the microbiota may make the environment of the Gastrointestinal system more sensitive to IBS. More studies are needed on the change of the gut microbiome concerning *D. fragilis* to gain a better understanding of the species involved and the potential interactions that they may have on the pathogenicity of *D. fragilis*.

## Future directions

With the current conflicting evidence over whether or not *D. fragilis* is a pathogen, we should no longer be asking the question, ‘Is *D. fragilis* a pathogen?’. Rather, we should be investigating ‘When is *D. fragilis* a pathogen?’, ‘How can we distinguish these pathogenic infections?’ and ‘What are the pathogenic pathways?’. It is unknown if this difference in clinical presentation is the result of undetected diversity of *D. fragilis* or if it is the result of currently unknown interactions with the gut microbiota [115]. A central requirement to all these studies is the need for rigorous diagnostics, and we encourage investigators to avoid the use of the lab-based assay, which has a number of serious pitfalls that undermine many current, published studies on *D. fragilis* [34,35].

While current molecular studies indicate that the population of *D. fragilis* is highly clonal, a limited number of genetic loci were studied, which limited their usefulness [57]. This limitation is due to the current lack of well-characterized genes and assembled genomic sequences, which would be helpful in identifying informative markers for future epidemiology studies. Additionally, a well-assembled and annotated genome could be key to resolving contradictions around the pathogenicity of *D. fragilis*. Clarification is also required on the life cycle and transmission pathway of *D. fragilis* to enable public health strategies to be developed to control the spread of this parasite.

## Abbreviations

IBS: irritable bowel syndrome
IFA: indirect fluorescence assay
MLG: multi-locus group
MLST: multi-locus sequence typing
qPCR: real-time PCR
TVV: *Trichomonas vaginalis* virus
